# Allele-specific methylation of the PSA promoter in prostate cells: A new translational marker for the differential diagnosis of prostate cancer

**DOI:** 10.1016/j.gendis.2024.101487

**Published:** 2024-12-09

**Authors:** Mikhail Baryshev, Irina Maksimova, Ilona Sasoveca

**Affiliations:** Institute of Microbiology and Virology, Riga Stradins University, Ratsupites Str 5, Riga LV 1067, Latvia

DNA methylation is one of the mechanisms of epigenetic control of gene expression, and a change in the intrinsic pattern can lead to various diseases and disorders. At the same time, this makes DNA methylation a disease-specific biomarker. For a long time, prostate-specific antigen (PSA) was used as an established tumor marker for prostate adenocarcinoma in the clinic for the diagnosis of prostate cancer (PCa).^1^ Despite the generally accepted fact that PSA is organ- but not cancer-specific, it still retains its diagnostic value for cancer detection.^2^ Here, we show that the acquisition of biallelic methylation status or even biallelic lack of methylation by the PSA promoter is a characteristic feature of cancer cells, while the monoallelic distribution of CpG/CCWGG methylation in the PSA promoter is a hallmark of noncancerous conditions. The coexisting CpG/CCWGG monoallelic methylation may indicate that CCWGG may represent a new signaling event for monoallelic methylation, suggesting novel functionality of this mark or representing benign prostatic hypertrophy conditions. In this study, we demonstrate that the methylation status of the non-CpG island PSA promoter spanning from −393 to +51 nucleotide positions, possessing 6 CpGs and 5 CCWGG epigenetic marks, has a distinctive methylation pattern in a PCa cell line model.

Exploring the altered status of DNA methylation to use it as a disease-specific biomarker, some researchers focus on CpG islands and do not attach importance to the methylation of several CpGs located in proximal promoters.^3^ However, it has been shown that methylation of even a single CpG in proximity to the transcription start site of *Pparg2* (peroxisome proliferator activated receptor gamma 2), a major regulator of adipogenesis, can silence the gene and thus prevent its expression.^4^ Bisulfite-treated DNA was used to determine the methylation of six CpG motifs and five CCWGG motifs that are targets of DNA methyltransferases (DNMTs) and therefore can epigenetically influence gene expression (Fig. 1A–D). SNP G-158A, located in the AREI of the PSA promoter, was considered in the genotyping of prostate cell lines for allele-specific methylation analysis (Fig. 1D). By genotyping prostate cell lines for SNP G-158A with sequencing of 20 clones, it was found that HPrEpiC, BPH1, LNCaP, PC3, and PA1 have the A/G, A/G, A/A, A/A and A/A genotypes, respectively. In the HPrEpiC cell line, only CpG methylation of the PSA promoter region was detected in a stochastic manner, with methylation detected in all 20 clones found in both alleles of the gene (Fig. 1E; Fig. S3). In contrast, the BPH1 cell line, in addition to CpG methylation, had two proximal CCWGGs methylated in the PSA promoter on the same allele (Fig. 1E; Fig. S4). We found that methylation of the two proximal CCWGG sites strictly distributed in the same allele coexisted with CpG methylation. Interestingly, the PA1 cell line, nonpermissive for PSA expression, also had the same methylation pattern with respect to CpG and CCWGG allelic methylation (Fig. 1E; Fig. S4). We hypothesize that the active allele in PA1 cells is properly silenced by DNA methylation, being imprinted, while there is a failure to imprint the correct allele in BPH1, and instead, the active allele is silenced, resulting in loss of PSA expression by the cells. Given the strict methylation of the two proximal CCWGG sites in the PSA promoter in PA1 and BPH1 cells, we suspect a novel pentanucleotide epigenetic mark functionality that may be related to the regulation of monoallelic gene expression. Looking at the 5′ flanking region of the PSA gene, in addition to the enhancer and proximal promoter, we also observed two regions, A and B, with clustered CpG/CCWGG sequences (Fig. 1B). PC3 and LNCaP, cancer cell lines, have a PSA promoter inverse methylation pattern: biallelically methylated and unmethylated, respectively (Fig. 1E; Fig. S1, 2). PC3 cells representing an aggressive PCa phenotype do not express PSA due to active allele silencing because both alleles are methylated, as we suspect. Clinically, this gene silencing can lead to false-negative results in PSA-based PCa testing; cancer cells will grow, but the PSA level will not increase. The LNCaP cell line, as an indolent form of the PCa phenotype, does indeed express PSA (Fig. 1F), demonstrating PSA promoter status as free from CpG and CCWGG methylation (Fig. 1E; Fig. S1).

**Figure 1:**
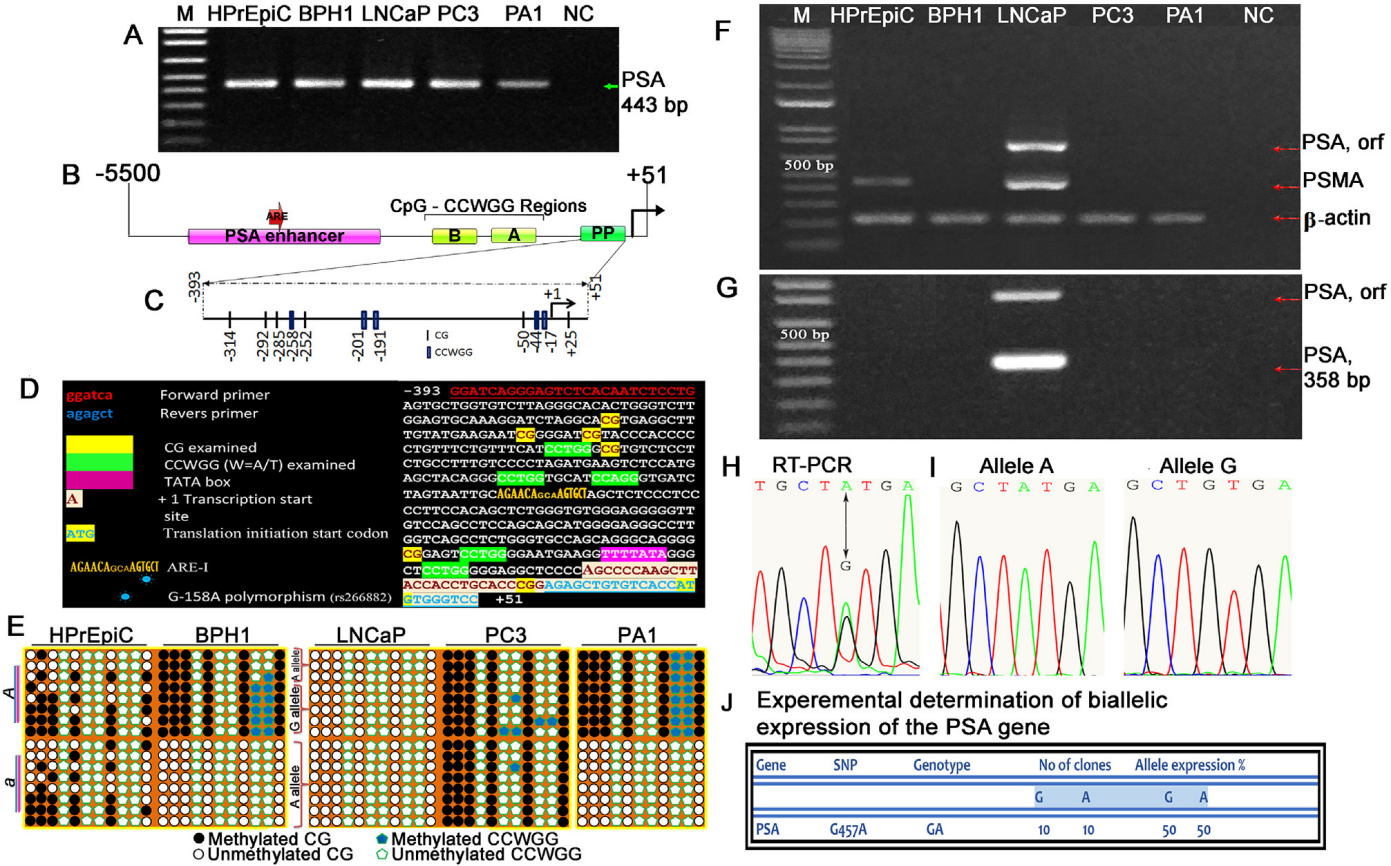
The proximal PSA promoter has an individual CG/CCWGG methylation pattern in prostate cell lines. **(A)** The gel photograph showing the amplification of the proximal region of the PSA promoter using BTD as a template and a pair of bisulfite-specific primers. **(B)** Schematic illustration of the 5′ flanking region of the PSA gene. The proximal promoter (PP) and the CG/CCWGG A and B regions are shown. **(C)** Schematic of the proximal PSA promoter. Short thin vertical lines indicate the position of the CG; short wide vertical lines represent CCWGG positions; an arrow indicates the transcription start site (TSS) (+1 bp). **(D)** Distribution of CpG and CCWGG, W = A/T) sites within the PP of the genomic sequence kallikrein-related peptidase 3 KLK3/PSA (Source: HGNC Symbol; Acc: HGNC: 6364; Chromosome 19: 50,854,915–50,860,764 forward strand). **(E)** Bisulfite sequencing analyses of the PSA PP. The status of PSA methylation in prostate cell lines and cells of non-prostatic origin was analyzed. *A* and *a* represent the parental alleles. At the bottom of the illustrations, the methylation status of the CpGs is shown. **(F–J)** PSA is biallelically expressed in LNCaP cells. (F) The gel photograph showing the result of RT-PCR that detects prostate-specific marker expression. (G) The gel photograph showing the result of nested PSA RT-PCR. The first round included amplification of the ORF PSA, and the second round used inner primers. (H) The chromatogram showing an A/G polymorphism in the coding region of the parental alleles, detected by RT-PCR ORF transcript sequencing, confirming cDNA heterozygosity. Two independent cDNA samples were used in cloning/sequencing experiments for RT-PCR products. (I) The chromatograms showing the A or G allele of individual clones that correspond to biallelic (A/G) PSA. (J) Experimental determination of biallelic expression of the PSA gene by cloning/sequencing.

None of the prostate cell lines, except for LNCaP, expressed PSA in any reverse transcription (RT) or nested RT-PCR analysis (Fig. 1F, G). PSMA (prostate-specific membrane antigen) expression was also detected to confirm the phenotypic “fingerprint” of the cell lines. Allele-linked single-nucleotide polymorphisms are useful features to estimate allelic expression in heterozygous genomes. We used an RT-PCR sequencing/cloning approach to detect polymorphisms in the transcribed region for subsequent assessment of PSA allele expression in the LNCaP cell line. The split peak in the chromatogram generated by sequencing the ORF PSA RT-PCR products indicates the existence of a heterozygous state of the PSA alleles (Fig. 1H). The GA polymorphism we identified at nucleotide 447, which is in exon 3, was further confirmed by sequencing independent clones (Fig. 1J). Sequencing of 20 clones of the full-length cDNA of PSA, transcript variant 1, revealed the presence of mRNA with G and A nucleotides distributed over 10 clones of each nucleotide variant, as shown in Figure 1I.

The mRNA expression of the three DNMTs was examined by quantitative RT-PCR in a PCa cell line model. DNMT1 and DNMT3B are equally expressed in BPH1 cells, and their profile is similar overall to that in PA1 cells, where monoallelic CG/CCWGG methylation of the PSA promoter occurs (Fig. S5). The level of DNMT1, which is responsible for maintaining the established methylation pattern, is significantly reduced (by 0.3 times) in the LNCaP cell line, where the PSA promoter is completely unmethylated, and conversely, the fully methylated promoter in PC3 cells is accompanied by an increase in the expression of DNMT1 (by 1.2 times), as shown in Figure S5. In addition, transcripts of DNMT3B, a *de novo* DNMT, are slightly down-regulated in LNCaP cells, reduced in PC3 cells, and heavily reduced in HPrEpiC cells that avoid CCWGG methylation. The expression profile of DNMTs in normal tissues shown in Figure S6, where DNMT1 shows a higher level of expression and DNMT3A intermediate and DNMT3B show a lower level of expression of PSA in the prostate, suggests that all studied cell lines have an altered pattern of DNMT expression compared with normal tissues.^5^

In conclusion, according to our study of prostate cancer cell lines, the translational ability of CG/CCWGG methylation in the PSA promoter to specific disease status in the clinic could be realized as follows: an elevated PSA level along with monoallelic promoter methylation would reflect benign prostatic hyperplasia or other benign conditions, elevated PSA levels due to biallelic expression of PSA and unmethylated PSA promoter status will be consistent with PCa. Low PSA levels may be accompanied by the growth of non-PSA-secreting cells of an aggressive phenotype with biallelic methylation of the PSA promoter. In other words, the determination of allele-specific methylation (Fig. S7) in the PSA promoter can be translated into a disease-specific state and can refine PSA-based PCa testing. Considering that clinical correlation studies are crucial to validate our results in real-world settings, we plan to conduct studies on clinical samples using local or international collaboration. Additional experiments to reduce DNMT1 expression in PC3 cells using pharmacological inhibitors or siRNA technology and overexpress DNMT3a in LNCaP cells will provide some insight into the actual mechanism leading to the somewhat unique methylation distribution. We propose to use purified Dnmt1 and Dnmt3a enzymes to methylate the PSA promoter PCR product using a separate DNMT1/DNMT3a reaction or their combined action as an alternative strategy. We suggest that these approaches will help elucidate the causal relationship between DNMT expression and PSA promoter methylation. It is important to note that the uniqueness of the discovered PSA methylation promoter may become a valuable marker for the authentication of prostate cell lines and can be widely used in laboratory practice.

## Author contributions

**Mihails Barisevs:** Conceptualization, Writing – original draft, Writing – review & editing. **Irina Maksimova:** Data curation, Writing – original draft. **Ilona Sasoveca:** Conceptualization, Data curation.

## Funding

The work was supported by the internal grant of the Rīga Stradiņš University (No. 132311).

## Conflict of interests

The authors declared no competing interests.

## References

[1] Stamey T.A., Yang N., Hay A.R., McNeal J.E., Freiha F.S., Redwine E. (1987). Prostate-specific antigen as a serum marker for adenocarcinoma of the prostate. N Engl J Med.

[2] Merriel S.W.D., Pocock L., Gilbert E. (2022). Systematic review and meta-analysis of the diagnostic accuracy of prostate-specific antigen (PSA) for the detection of prostate cancer in symptomatic patients. BMC Med.

[3] Olkhov-Mitsel E., Van der Kwast T., Kron K.J. (2012). Quantitative DNA methylation analysis of genes coding for kallikrein-related peptidases 6 and 10 as biomarkers for prostate cancer. Epigenetics.

[4] Baryshev M., Petrov N., Ryabov V., Popov B. (2022). Transient expression of inactive RB in mesenchymal stem cells impairs their adipogenic potential and is associated with hypermethylation of the PPARγ2 promoter. Genes Dis.

[5] Fagerberg L., Hallström B.M., Oksvold P. (2014). Analysis of the human tissue-specific expression by genome-wide integration of transcriptomics and antibody-based proteomics. Mol Cell Proteomics.

