# Supplementary material for: Allele-specific methylation of the PSA promoter in prostate cells: A new translational marker for the differential diagnosis of prostate cancer
Source: Genes Dis. 2024 Dec 9;12(3):101487. doi: 10.1016/j.gendis.2024.101487 (PMC11804549; doi:10.1016/j.gendis.2024.101487)
Supplement: Multimedia component 1 [file mmc1.pdf]

Supplementary material

Baryshev Mikhail1*, Maksimova Irina, Sasoveca Ilona

Institute of Microbiology and Virology, Riga Stradins University, Ratsupites Str 5, LV-1067, Riga, Latvia;

*Corresponding author: E-mail: Mihails.Barisevs@rsu.lv

E-mail: Mihails.Barisevs@rsu.lv; Telephone +37128280853

Materials and Methods.

Cell lines

Human prostatic carcinoma cell lines PC3 (ATCC® CRL1435™) and LNCaP (ATCC CRL-1740™) were purchased from the American Type Culture Collection (ATCC); BPH1 (DSMZ no.: ACC 143) from the DSMZ, German Collection of Microorganisms and Cell Cultures, GmbH and the human prostatic epithelial cell line (HPrEpiC) were obtained from the ScienCell Research Laboratories. All cell lines were cultured at 37 °C in a humidified incubator with 5% CO_2 according to the manufacturer's protocol.

DNA extraction and bisulfite treatment

Genomic DNA was obtained by overnight cell incubation in TES buffer containing 0.1% SDS and 100 µg/ml proteinase K at 55°C with subsequent phenol/chloroform extraction and isopropanol precipitation. Two micrograms of DNA in 50 µl of TE buffer was denatured for 15 min in 0.3 M NaOH at 37 °C. The denatured DNA was mixed with 550 µl of freshly prepared solution of 10 mM hydroquinone and 3 M sodium bisulfite at pH 5.0 and incubated under mineral oil at 50°C for 12 h. Bisulfite-treated DNA was desalted by isopropanol precipitation, desulfonated with 0.3 M NaOH for 5 min at room temperature, and precipitated with ethanol. Converted DNA was dissolved in 100 µl of TE and stored at -20°C. Three microliters of precipitated DNA was used for each PCR.

Bisulfite sequencing analysis

Bisulfite-treated DNA (BTD) was used to amplify the proximal region of the PSA promoter with primers specific for bisulphite-converted DNA listed in Table 1.

The PCR was carried out using 2.5 units of homemade Taq polymerase in a final volume of 50 µl and the following cycling conditions: 5 minutes at 95 °C, followed by 35 cycles (30 seconds denaturation at 95 °C, annealing for 30 seconds at 62 °C and elongation at 72 °C for 1 minute). The PCR products were gel purified and cloned using a TOPO TA cloning kit. To prevent clonal amplification of sequences, the competent cells transformed were plated immediately after heat

shock, excluding shaking bacteria for 1 h. Plasmid DNA of individual clones was purified with the DM method[6]. A total of 18-20 clones for PCR products were sequenced and analyzed. Promoter methylation analysis was performed by aligning sequenced clones with the PSA promoter region, where cytosine to uracil was converted in an in silico experiment. The efficiency of cytosine to uracil conversion was estimated as the ratio of cytosine in a non-CpG context to the total number of cytosines in the region. The clones with an efficiency of cytosine conversion less than 98% were omitted from the analysis. DNA sequencing was performed using the ABI BigDye Terminator Cycle Sequencing Kit v3.1 according to the manufacturer's instructions on a Gene Amp 9700 PCR machine, and the sequences were detected on an ABI 3130XL Genetic Analyzer.

RNA extraction

Total RNA was extracted from HPrEpiC, BPH-1, LNCaP, PC3 and PA1 cells using TRIzol reagent (Invitrogen, California, USA) following the supplier's instructions. The extracted RNA was dissolved at 55 °C in sterile water treated with diethylpyrocarbonate (DEPC) (Sigma, St. Louis, USA). The concentration of the total RNA was measured with a Qubit Fluorometer (Invitrogen, Carlsbad, CA, USA), and RNA integrity was evaluated through 1% agarose gel electrophoresis to visualize a certain banding pattern and by amplification of the housekeeping gene. The resulting PCR product was subjected to gel electrophoresis in 1% agarose and visualized by ethidium bromide staining and UV transillumination.

cDNA synthesis, RT/Nested RT–PCR

Five micrograms was reverse transcribed into cDNA using the Maxima H Minus First Strand cDNA synthesis kit with ds DNase (Thermo Fisher Scientific Inc. Waltham, MA, USA) according to the manufacturer's instructions. PSA and PSMA expression as specific markers of prostate cell lines was estimated by RT–PCR. Two microliters of the resulting PSA PCR product was subjected to a second nested PCR using the primers listed in Table 1. Each cDNA sample was assayed in duplicate using primers for both PSA and PSMA. The fidelity of amplification

for the nested primer products was confirmed by sequencing and was found to be consistent within one base pair of the PSA reference sequences.

Quantitative real-time PCR

To quantify the level of DNMT expression, cDNA was amplified with the CFX96™ Real-Time PCR detection system (Bio-Rad Laboratories Inc., Richmond, CA, USA) and PerfeCTa SYBR Green FastMix (Quanta BioSciences Inc., Beverly, MA, USA). Built-in data analysis modules with automatic baseline subtraction and threshold setting of CFX manager were used to analyze the normalized (ΔΔCT) DNMT mRNA expression. TATA box-binding protein (TBP), the housekeeping gene, was used to obtain relative normalized mRNA expression**.** Melting curve analysis was performed ramping from 60 °C to 90 °C and rising by 0.5 °C every 2 s. The relative expression levels were calculated using the $2^{-\Delta\Delta CT}$ method and CFX manager software version 3.0. All experiments were conducted in triplicate.

Primer design

The primer-BLAST software tool was used to design new target-specific primers in RT–PCR experiments. This tool is available at http://www.ncbi.nlm.nih.gov/tools/primer-blast. All amplicon primers were designed to encompass exon–exon boundaries to avoid genomic DNA amplification. The specificity of amplicons and primer pairs was checked in silico using BLAST (National Center for Biotechnology Information) alignment tools.

To design primer-to-bisulphite-region-specific matches for bisulfite-modified DNA, the following rule was applied: since the DNA strands are no longer complementary after bisulfite treatment, an individual primer set will only amplify one strand of the target sequence. The first primer was designed to anneal to the converted target sequence. The second primer was designed to anneal to the extension product of the first primer, not the opposite template strand. The PrimerSuite program (PrimerSuite, PrimerDimer and PrimerPlex) was used to generate robust primers for PCR bisulfite sequencing analysis. The PrimeSuite, PrimerDimer, and PrimerPlex

modules are all available online at www.primersuite.com, www.primer-dimer.com, and www.primer-plex.com, respectively.

Bioinformatics

National Centre for Biotechnology Information (NCBI) database resources for bioinformatics have been used to assess DNMT expression in normal prostate tissue. The Basic Local Alignment Search Tool (BLAST) commonly used in bioinformatics has been applied to search for similarities and identify homologous sequences. The Genotype-Tissue Expression (GTEx) project data of Gene Expression in 54 tissues from GTEx RNA-seq of 17382 samples, 948 donors (V8, Aug 2019) and RNA expression level in 33 TCGA Cancer Tissues (GENCODE v23) produced by the consortium were used with UCSC Genome Browser on Human (GRCh38/hg38). The TCGA chose cancers for study based on two broad criteria: poor prognosis/overall public health impact and availability of human tumor and matched normal tissue samples that meet TCGA standards.

Statistical methods and associated software

The CFX96™ Real-Time PCR detection system associated software and algorithm were used in this study.

Table 1 Primer sequences for RT, RT–qPCR and bisulfite sequencing analysis.

RT–qPCR,

RT–PCR

Gene Symbol	Sequence (5'⟶ 3') Forward/Reverse	Exon Location	Tm(°C)	Size (bp)	Accession No.
DNMT1	AGACCATCAGGCATTCTACCA	4	60	135	NM_001160045
	CGTTCTCCTTGTCTTCTCTGT	6			
DNMT3A	TATTGATGAGCGCACAAGAGAGC	11	60	136	NM_022552
	TTGGCACATTCCTCCAACGAAG	12			
DNMT3B	GAATTACTCACGCCCCAAGGA	19	60	135	NM_006892
	TGGCATCAATCATCACTGGATTAC	20			
TBP	GAGCCAAGAGTGAAGAACAGTCC	5, 6	60	130	NM_003194
	AACTTCACATCACAGCTCCCCA	6			
PSA ORF	AGCTGTGTCACCATGTGGG	1	60	799	NM_001648
	CTCAGGGGTTGGCCACGA	5			
PSA Inner	CAGTCTGCGGCGGTGTTCT	2	58	358	NM_001648
	GGGTCAAGAACTCCTCTGGTTCA	3,4			
PSMA	GAAACTGGACCCCAGGTCTGGA	1	60	478	NM_004476
	CAGGCCAAATTCTTTCCACTGGGA	3			
ACTB	CGCCCTGCCTATCTGTATT	4	60	230	NM_001101.5
	TCCCCACAGGGAGTGTGTAG	5			

Bisulphite sequencing					
Gene Symbol	Sequence (5'⟶ 3') Forward/Reverse	Promoter Location	Tm (°C)	Size (bp)	Accession No.
PSA	GGATTAGGGAGTTTTATAATTTTTTG	-393	64	443	Acc:HGNC:6364
	AAACCCACATAATAACACAACTCT	+51			50,854,915-50,860,764

Supplementary figures

Fig. S 1 Representative chromatogram of the PSA promoter without CG methylation, specific for both the LNCaP alleles and one of the parental BPH1 alleles.

Fig. S 2 Representative chromatogram of complete CG methylation of the PSA promoter specific for both PC3 alleles. Methylated CG is depicted with a pictogram.

Fig. S 3 Representative chromatogram of partial CG methylation of the PSA promoter specific for both HPrEpiC alleles. Methylated CG is depicted with a pictogram.

Fig. S 4 Representative chromatogram of CG/CCWGG methylation of the PSA promoter specific for one of the parental BPH1and PA1 alleles. Methylated CG is depicted with a pictogram.

Fig. S5 DNMT profile in cells with PSA monoallelic methylation is similar overall. DNMT1 is downregulated in LNCaP cells with an unmethylated PSA promoter. In contrast, it is activated in PC3 cells, where the PSA promoter is biallelically methylated. The relative expression of DNMT1, DNMT3A and DNMT3B mRNA was assessed by qRT–PCR. The results were normalized to that of TBP expression. All data are presented as the mean ± *SD* of three independent experiments.

Fig. S6 DNMT expression levels determined by RNA sequencing in normal prostate tissues. RNA sequencing was performed on tissue samples from 95 people. Image adapted from NCBI Bioinformatics Database resources: BioProject, PRJEB4337 [5].

Fig. S7 Conceptual diagram of gene expression mediated by methylation.

The two parental alleles of a gene are designated "*A*" and "*a*". Depending on acquired epigenetic information (methylation state of alleles), gene expression will be realized as follows: monoallelic expression occurs when only the active allele is transcribed, retaining the unmethylated status. Which of the two alleles is transcribed may depend on each allele’s parental origin, or an allele may be selected at random. When both alleles are no longer methylated, both alleles are transcribed. In the case of biallelic methylation, transcription is abolished. The monoallelic arrangement of CG/CCWGG methylation suggests the ability of the CCWGG mark to serve as a secondary imprint if not present in gDMR, which is known as a primary imprint.
