# Supplementary material for: Allele-specific methylation of the PSA promoter in prostate cells: A new translational marker for the differential diagnosis of prostate cancer
Source: Genes Dis. 2024 Dec 9;12(3):101487. doi: 10.1016/j.gendis.2024.101487 (PMC11804549; doi:10.1016/j.gendis.2024.101487)

Supplementary figures

Sequencing of DM miniprepped DNA applied for proximal PSA promoter methylation analysis.

Supplementary Fig. S 1 Representative chromatogram of the PSA promoter without CG methylation, specific for both the LNCaP alleles and one of the parental BPH1 alleles.

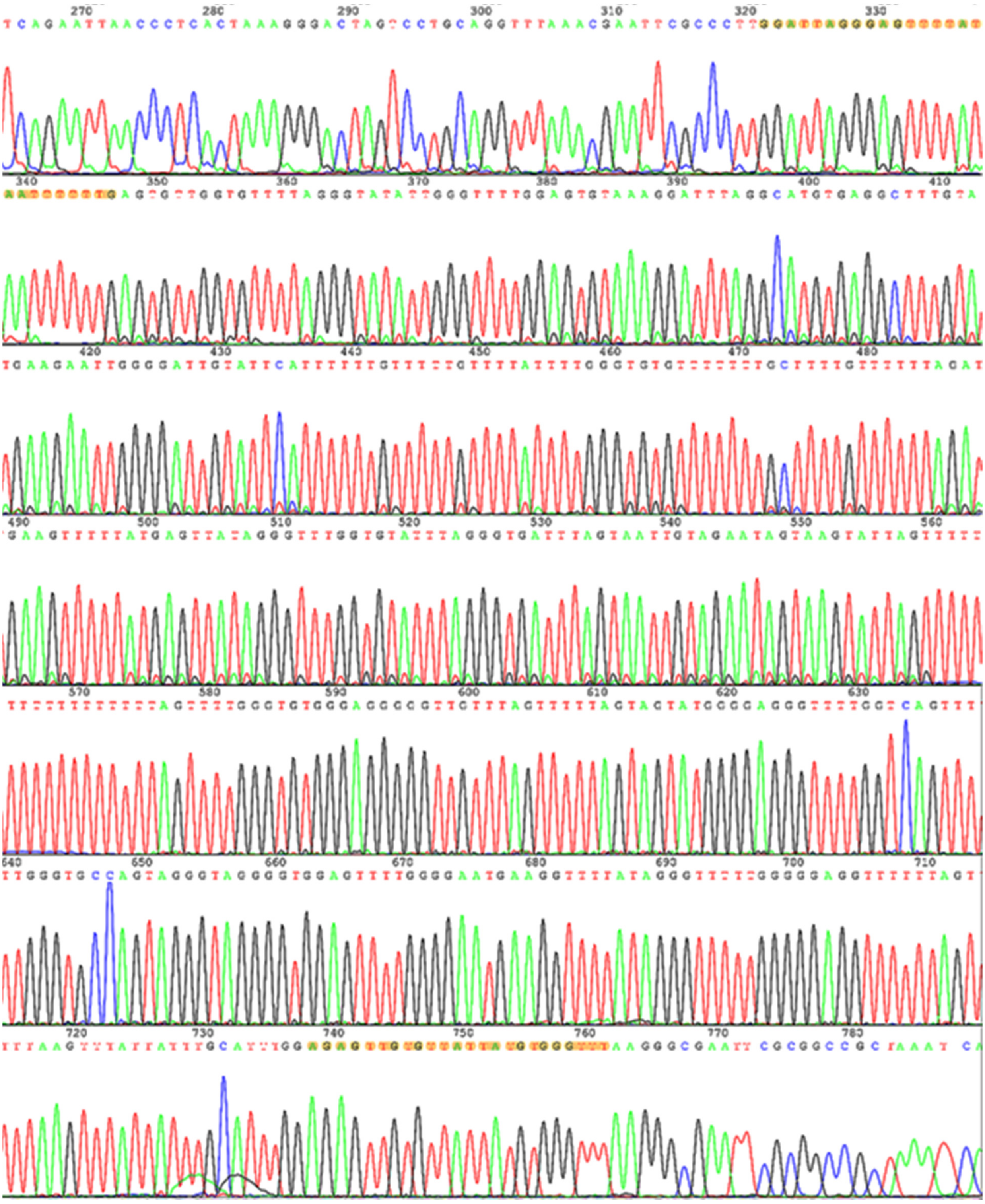

Supplement: Multimedia component 2 [file mmc2.pdf]
