# Supplementary material for: Allele-specific methylation of the PSA promoter in prostate cells: A new translational marker for the differential diagnosis of prostate cancer
Source: Genes Dis. 2024 Dec 9;12(3):101487. doi: 10.1016/j.gendis.2024.101487 (PMC11804549; doi:10.1016/j.gendis.2024.101487)

Supplementary Fig. S 3 Representative chromatogram of partial CG methylation of the PSA promoter specific for both HPrEpiC alleles.

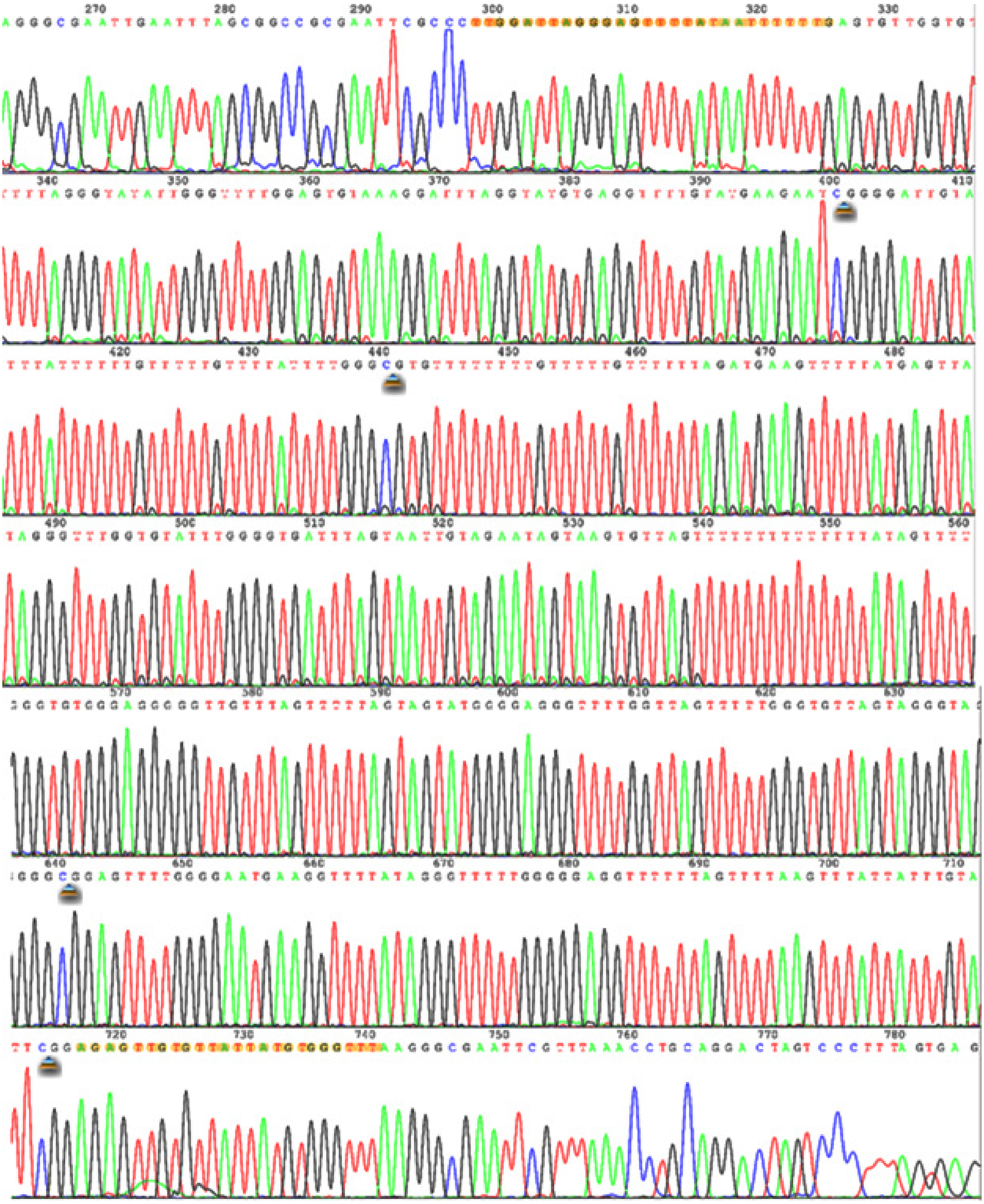

Supplement: Multimedia component 4 [file mmc4.pdf]
