# Supplementary material for: Allele-specific methylation of the PSA promoter in prostate cells: A new translational marker for the differential diagnosis of prostate cancer
Source: Genes Dis. 2024 Dec 9;12(3):101487. doi: 10.1016/j.gendis.2024.101487 (PMC11804549; doi:10.1016/j.gendis.2024.101487)

Supplementary Fig. S 4 Representative chromatogram of CG/CCWGG methylation of the PSA promoter specific for one of the parental BPH1and PA1 alleles.

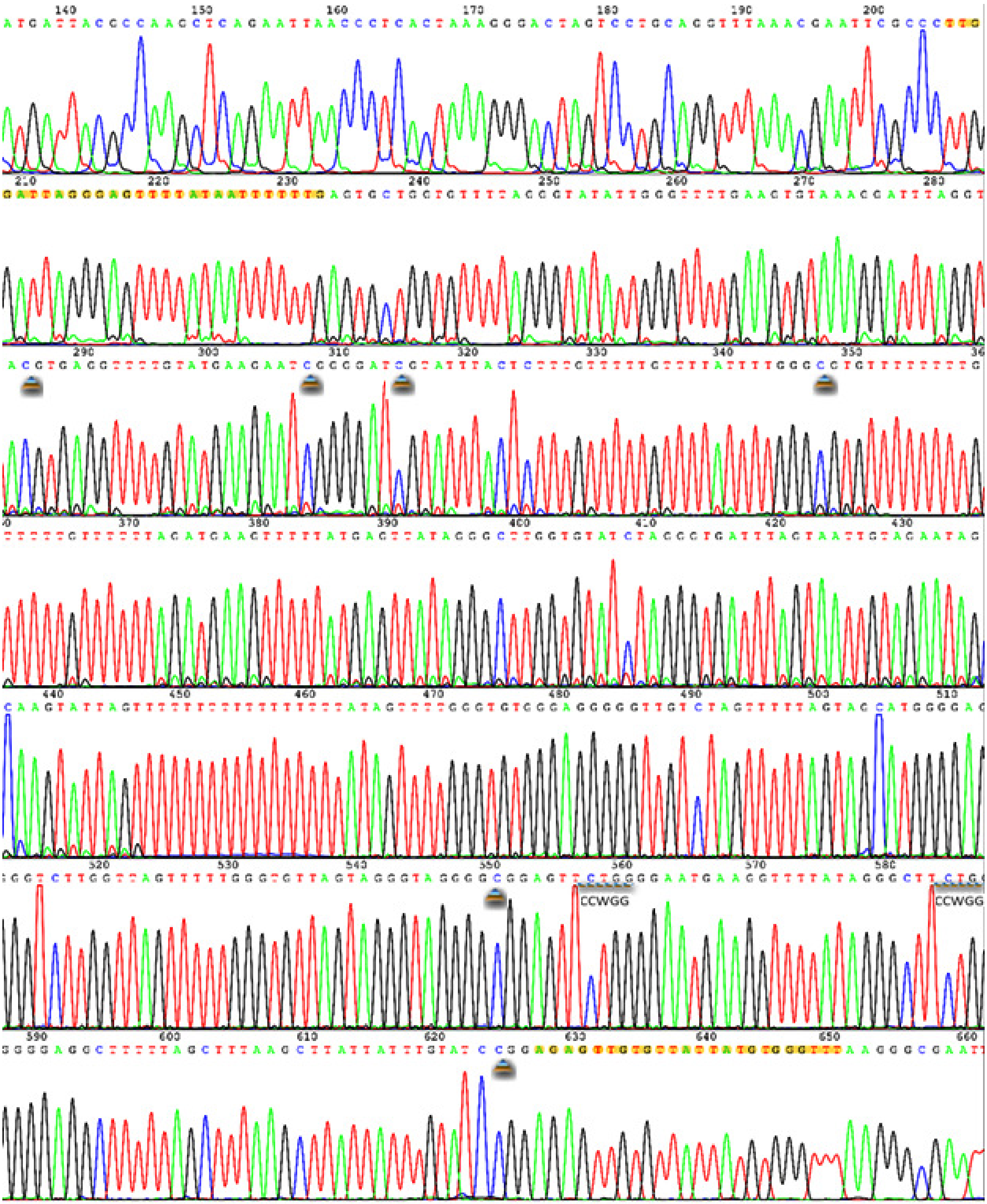

Supplement: Multimedia component 5 [file mmc5.pdf]
