# Supplementary material for: Allele-specific methylation of the PSA promoter in prostate cells: A new translational marker for the differential diagnosis of prostate cancer
Source: Genes Dis. 2024 Dec 9;12(3):101487. doi: 10.1016/j.gendis.2024.101487 (PMC11804549; doi:10.1016/j.gendis.2024.101487)

Supplementary Fig. S 5 DNMT profile in cells with PSA monoallelic methylation is similar overall. DNMT1 is downregulated in LNCaP cells with an unmethylated PSA promoter. In contrast, it is activated in PC3 cells, where the PSA promoter is biallelically methylated. The relative expression of DNMT1, DNMT3A and DNMT3B mRNA was assessed by qRT–PCR. The results were normalized to that of TBP expression. All data are presented as the mean ± SD of three independent experiments.

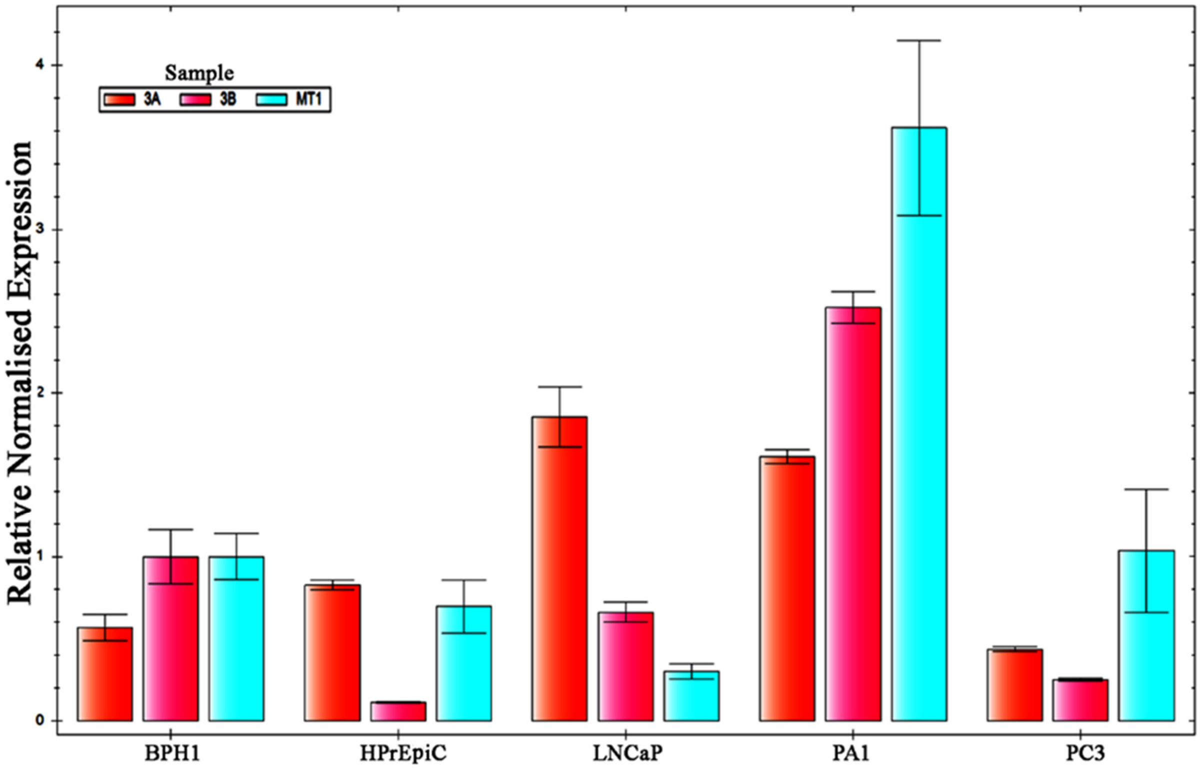

Supplement: Multimedia component 6 [file mmc6.pdf]
