# Supplementary material for: Allele-specific methylation of the PSA promoter in prostate cells: A new translational marker for the differential diagnosis of prostate cancer
Source: Genes Dis. 2024 Dec 9;12(3):101487. doi: 10.1016/j.gendis.2024.101487 (PMC11804549; doi:10.1016/j.gendis.2024.101487)

Supplementary Fig. S 6 DNMT expression levels determined by RNA sequencing in normal prostate tissues. RNA sequencing was performed on tissue samples from 95 people. Image adapted from NCBI Bioinformatics Database resources: BioProject, PRJEB4337[5].

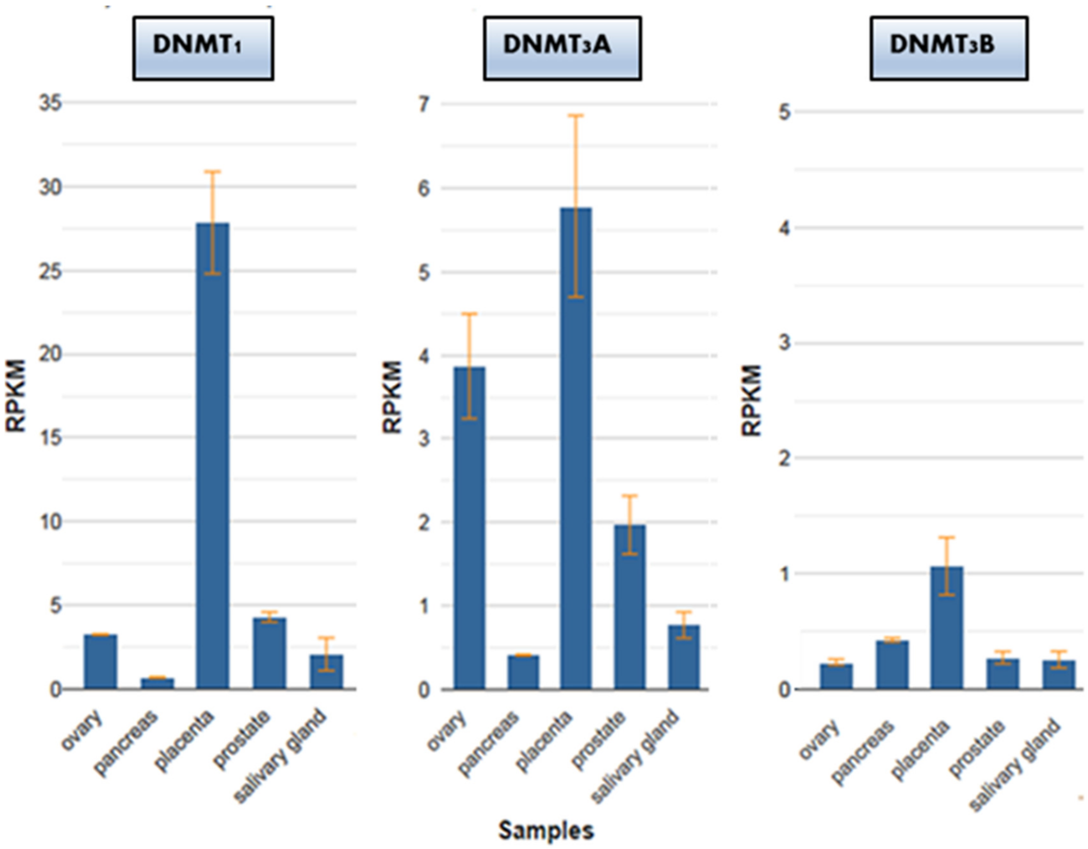

Supplement: Multimedia component 7 [file mmc7.pdf]
