# Supplementary material for: Allele-specific methylation of the PSA promoter in prostate cells: A new translational marker for the differential diagnosis of prostate cancer
Source: Genes Dis. 2024 Dec 9;12(3):101487. doi: 10.1016/j.gendis.2024.101487 (PMC11804549; doi:10.1016/j.gendis.2024.101487)
Supplement: Multimedia component 8 [file mmc8.pdf]

Supplementary Fig. S 7 Conceptual diagram of gene expression mediated by methylation. The two parental alleles of a gene are designated "A" and "a". Depending on acquired epigenetic information (methylation state of alleles), gene expression will be realized as follows: monoallelic expression occurs when only the active allele is transcribed, retaining the unmethylated status. Which of the two alleles is transcribed may depend on each allele's parental origin, or an allele may be selected at random. When both alleles are no longer methylated, both alleles are transcribed. In the case of biallelic methylation, transcription is abolished. The monoallelic arrangement of CG/CCWGG methylation suggests the ability of the CCWGG mark to serve as a secondary imprint if not present in gDMR, which is known as a primary imprint.

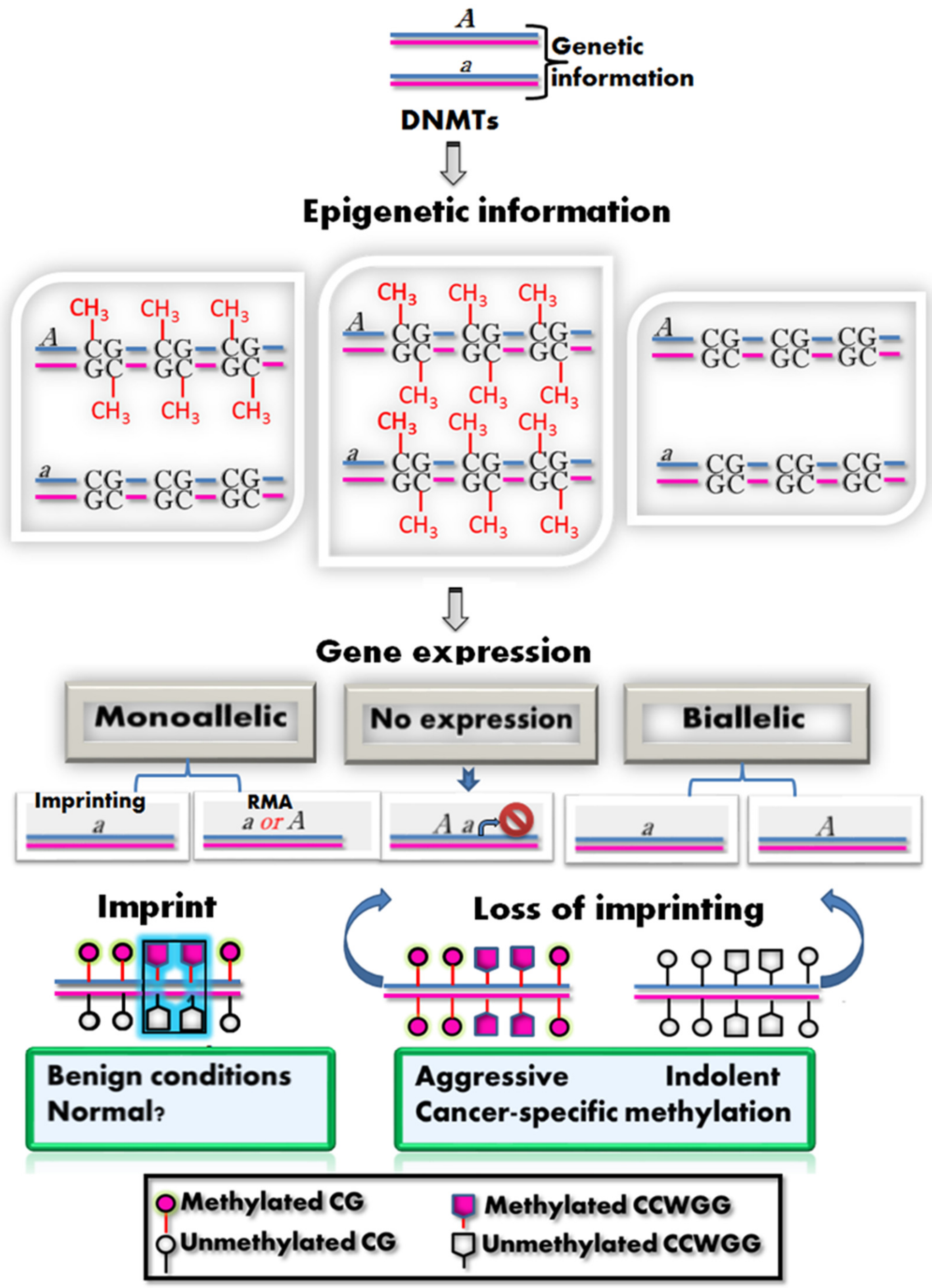

A
a
Genetic information
DNMTs
Epigenetic information
Gene expression
Monoallelic
No expression
Biallelic
Imprinting
RMA
a or A
Imprint
Loss of imprinting
Benign conditions
Normal?
Aggressive
Indolent
Cancer-specific methylation
Methylated CG
Unmethylated CG
Methylated CCWGG
Unmethylated CCWGG
